# Adjuvant-induced arthritis promotes vascular hyporesponsiveness to phenylephrine through a nitric oxide-related mechanism

**DOI:** 10.1590/1414-431X2024e13304

**Published:** 2024-05-17

**Authors:** T.S. Araujo, M.A. Spadella, C.P. Carlos, C.R. Tirapelli, E.F.B. Chagas, J.C.D. Pinheiro, A.B. Chies

**Affiliations:** 1 Laboratório de Farmacologia, Faculdade de Medicina de Marília, Marília, SP, Brasil; 2 Laboratório de Embriologia Humana, Faculdade de Medicina de Marília, Marília, SP, Brasil; 3 Laboratório de Pesquisa Experimental, Faculdade de Medicina Faceres, São José do Rio Preto, SP, Brasil; 4 Disciplina de Fisiologia, Faculdade de Medicina de Marília, Marília, SP, Brasil; 5 Laboratório de Farmacologia Cardiovascular, Escola de Enfermagem de Ribeirão Preto, Universidade de São Paulo, Ribeirão Preto, SP, Brasil; 6 Centro Interdisciplinar de Diabetes, Universidade de Marília, Marília, SP, Brasil; 7 Programa de Mestrado Interdisciplinar em Interações Estruturais e Funcionais em Reabilitação, Universidade de Marília, Marília, SP, Brasil; 8 Programa de Mestrado em Saúde e Envelhecimento, Faculdade de Medicina de Marília, Marília, SP, Brasil

**Keywords:** Experimental arthritis, Cardiovascular system, Vascular endothelium, Vasoconstriction, Nitric oxide

## Abstract

Arthritis has important cardiovascular repercussions. Phenylephrine-induced vasoconstriction is impaired in rat aortas in the early phase of the adjuvant-induced arthritis (AIA), around the 15th day post-induction. Therefore, the present study aimed to verify the effects of AIA on hyporesponsiveness to phenylephrine in rat aortas. AIA was induced by intradermal injection of *Mycobacterium tuberculosis* (3.8 mg/dL) in the right hind paw of male Wistar rats (n=27). Functional experiments in isolated aortas were carried out 15 days after AIA induction. Morphometric and stereological analyses of the aortas were also performed 36 days after the induction of AIA. AIA did not promote structural modifications in the aortas at any of the time points studied. AIA reduced phenylephrine-induced contraction in endothelium-intact aortas, but not in endothelium-denuded aortas. However, AIA did not change KCl-induced contraction in either endothelium-intact or denuded aortas. L-NAME (non-selective NOS inhibitor), 1400W (selective iNOS inhibitor), and ODQ (guanylyl cyclase inhibitor) reversed AIA-induced hyporesponsiveness to phenylephrine in intact aortas. 7-NI (selective nNOS inhibitor) increased the contraction induced by phenylephrine in aortas from AIA rats. In summary, the hyporesponsiveness to phenylephrine induced by AIA was endothelium-dependent and mediated by iNOS-derived NO through activation of the NO-guanylyl cyclase pathway.

## Introduction

Rheumatoid arthritis (RA) is a chronic, autoimmune, debilitating disease, which is characterized by inflammation and articular damage. The disease affects 1% of the world's adult population, with a higher incidence in the elderly (1). RA first and most severely affects the joints, where a persistent inflammation of the synovial membrane is observed. Moreover, synovial fibroblasts and macrophages promote joint destruction and the production of pro-inflammatory cytokines, which favor chronic inflammation (2,3).

Cytokines and other inflammatory mediators, produced in higher levels in the joints affected by RA, can enter the bloodstream and spread systemically (4). In this manner, RA can trigger extra-articular manifestations, including cardiovascular manifestations, which can lead to a 50% increase in mortality among these patients (4,5).

The cardiovascular manifestations of RA usually coincide with endothelial dysfunction, which apparently results from oxidative stress caused by the action of joint-derived pro-inflammatory cytokines on the vascular endothelium (4). Reactive oxygen species (ROS), superoxide in particular, react with endothelium-derived nitric oxide (NO), producing peroxynitrite (6). This process leads to an increased response to several vasoconstrictor agonists, besides promoting pro-inflammatory and pro-thrombotic action in the vasculature (7).

In contrast, impairment contractile aorta responses to α_1_-adrenergic agonists have also been observed in rats submitted to adjuvant-induced arthritis (AIA) (8,9). In these studies, impairments in the contractile responses of the aorta occurred in the initial phase of the model, i.e., in the preclinical phase (8), or up to 30 days after AIA induction (9). This vascular manifestation of arthritis has been much less investigated, and thus, its pathophysiological mechanism is still poorly understood. It is believed that joint-derived pro-inflammatory cytokines may act in aortas, leading to increased expression of inducible nitric oxide synthase (iNOS) (10). Thus, there is an increase in the local synthesis of NO that, in turn, leads to impairment in contractile responses. Similarly, a decreased response of rat knee joint blood vessels to phenylephrine was observed following adjuvant-induced joint inflammation, which involved NO (11).

Thus, the present study aimed to verify the effects of AIA on the responses of rat aortas to phenylephrine, focusing on the participation of NO-related mechanisms.

## Materials and Methods

### Animals

Male Wistar rats (n=54; 12 weeks old) were housed in standard polyethylene cages (50×40×20 cm), with three animals per cage, in an environment with controlled temperature (21-24°C) and lighting (12/12 h), with free access to food and water. The Research Ethics Committee of the Marilia Medical School (CEUA-Famema; protocol No. 2935/20) approved this study.

### Adjuvant-induced arthritis protocol

AIA was induced by administering modified Freund's Complete Adjuvant, composed of 100 μL of mineral oil and distilled water-based emulsion containing *Mycobacterium tuberculosis* H37RA at 3.8 mg/mL (BD Difco™ Adjuvants, USA) via the intradermal route into the sole of the animal's right hind paw (n=27). The control animals (n=27) received only an equal volume of mineral oil by the same route of administration. On the day of AIA induction and 15 and 36 days later, body weight and the diameter of the right and left hind legs (tibiotarsal region - using a pachymeter) were measured.

### Animal euthanasia and sample collection

On day 15 or 36 after the induction of AIA, the animals were euthanized by carbon dioxide (CO_2_) inhalation, followed by exsanguination. Then, the aortas were removed, separated from the adjacent tissues, and washed in saline solution. Part of them was fixed in 4% paraformaldehyde in PBS, pH 7.2, for at least 24 h for morphometric, stereological, and immunohistochemical analyses. The other part was immediately assigned to functional studies in an organ bath or used for the determination of nitrite/nitrate.

### Morphometric and stereological analysis

After fixation, the aorta segments were washed in running water for 24 h and then immersed in 70% alcohol solution until processing for inclusion. A part of these segments was included in resin for later morphometric analysis. For this, the samples were dehydrated in 95% ethyl alcohol and embedded in methacrylate-glycol resin (Leica Historesin - Embedding Kit, USA). The blocks were sectioned on a Leica RM2245 microtome to obtain 5-µm-thick sections, which were stained with hematoxylin and eosin (HE). Then, panoramic images of the aorta of each animal were photomicrographed for subsequent measurement of the total thickness (µm) of the vessel and media layer in 4 different histological fields, obtained in the same histological section using CellSens Standard software (Olympus, Japan).

The remaining aorta samples were dehydrated in increasing concentrations of alcohol, diaphanized in butyl alcohol, infiltrated, and embedded in histological paraffin (Synth) for analysis of total collagen. Sections of 5 µm thickness were also obtained from these blocks and stained with Masson trichrome. For quantification of total collagen, 7 to 12 histological fields per animal were captured using an Olympus DP-25 digital camera attached to an Olympus BX41 microscope (Olympus). The number of blue dots was counted using the Olympus CellSens by Dimension software with the “manual threshold adjustment” and “count” tools.

In both analyses, the results corresponded to the average of the measurements obtained in the different histological fields of each animal.

### Immunohistochemical analysis

Slides with sections of paraffin-embedded samples obtained from the animals on the 15th day after induction were first deparaffinized, submitted to the antigen exposure process by incubation in citrate buffer (0.01 mol/L, pH 6.0), and blocking of endogenous peroxidase with methanol and 3% hydrogen peroxide. Non-specific binding of proteins was blocked by prior incubation with 1% bovine serum albumin (BSA) for 10 min in phosphate-buffered saline (PBS), pH 7.4. Next, without washing the fragments, we proceeded with overnight incubation at 4°C with anti-NOS2 monoclonal antibody (1:80) (SC-7271; Santa Cruz Biotechnology, USA). Negative controls were incubated with a 1% concentration equivalent of PBS-BSA instead of primary antibody (reaction control). Upon completion, the fragments were incubated with streptavidin-conjugated, anti-mouse secondary antibody (1:1000) (E-AB-1001, Elabscience Biotechnology Inc., USA). Staining was performed with DAB (3,3-diaminobenzidine, Invitrogen DAB kit, USA) for 10 min and counterstained with hematoxylin for 2 min, dehydrated, and prepared. Finally, these slides were photomicrographed in 4 histological fields using an Olympus DP-25 digital camera attached to an Olympus BX41 microscope (Olympus). The number of marked points in the immunolabeled regions (brown staining) was determined by the Olympus CellSens by Dimension software, using the “manual threshold” and “count” tools. The results are reported as the mean number of iNOS-positive cells in the different histological fields per experimental group.

### Study of vascular reactivity

Thoracic aortas isolated from animals on day 15 post-induction were sectioned into 3-mm rings in a paraffin-coated Petri dish. Then, the rings were placed in organ baths with a capacity of 2 mL between 2 metal hooks (one of them attached to the bottom of the bath and the other connected to an isometric force transducer). The rings were kept under the tension of 1.5 *g* for 60 min, immersed in Krebs-Henseleit nutrient solution (composition in mmol/L: 130.0 NaCl; 4.7 KCl; 1.6 CaCl_2_; 1.2 KH_2_PO_4_; 1.2 MgSO_4_; 15.0 NaHCO_3_; and 11.1 glucose), with pH adjusted to 7.4, bubbled with the carbogenic mixture (95% O_2_ and 5% CO_2_) and heated to 37°C. The changes in tone of these preparations were recorded using a Powerlab 8/30 data acquisition system (AD Instruments, Australia).

Then, the preparations were stimulated three times with 90 mM KCl, and endothelial integrity was functionally tested by stimulation with acetylcholine (10^-5^ mol/L). In preparations whose endothelium was mechanically removed, acetylcholine-induced relaxation was not observed. Acetylcholine-induced relaxation greater than or equal to 50% of phenylephrine-induced precontraction was considered indicative of endothelial integrity.

Endothelium-intact or denuded aortic rings were stimulated with cumulative concentrations of phenylephrine (10^-10^ to 10^-4^ mol/L). In the case of intact preparations, these stimulations were made in both the absence and presence of 10^-4^ mol/L L-NAME (non-selective NOS inhibitor), 10^-6^ mol/L 1400W (selective iNOS inhibitor), 10^-4^ mol/L 7-nitroindazole (7-NI, selective nNOS inhibitor), 10^-6^ mol/L yohimbine (selective α_2_-adrenergic receptor antagonist), 10^-5^ mol/L indomethacin (non-selective COX inhibitor), and 10^-6^ mol/L 1H-(1,2,4)-oxadiazole-[4,3-a]-quinoxaline-1-one (ODQ, selective guanylyl cyclase inhibitor). These drugs were directly administered in the organ bath 20 min before stimulations. Some intact preparations were also stimulated with KCl (1.2×10^-2^ mol/L, compensated by reducing the Na+ concentration).

The aortic vasomotor responses were recorded graphically as concentration-response curves, from which the pEC50 - negative of the logarithm of the molar concentration of the agonist responsible for 50% of the maximum effect (EC50) - was obtained. This parameter was calculated by non-linear regression using Prisma 6.0 software (GraphPad Software Corp., USA). The maximum contractile responses (Emax), elicited by supramaximal agonist concentrations, were also determined.

### Nitrite/nitrate determination

According to the method adapted from the study by Carda et al. (12), aorta segments (5 mm) obtained from animals on day 15 after induction were homogenized in 200 µL of PBS, pH 7.2, and centrifuged at 2,195 *g* for 10 min at 4°C. The supernatant was ultrafiltered at 2,195 *g* for 10 min at 4°C. Then, 50 µL aliquots of the sample were transferred to Eppendorf tubes containing 250 µL of 20 mmol/L phosphate buffer, 25 µL of 1.8 µmol/L NADPH, and 25 µL of 1 U/mL nitrate reductase enzyme, followed by incubation for 1 h and 30 min at 37°C. After this period, 25 µL of 80 µmol/L phenazine methosulfate was added and followed by homogenization and incubation in the dark for 30 min. Zinc sulfate (120 µL, 0.5 mol/L) diluted in 50% ethanol and 120 µL of 0.5 mol/L Na_2_CO_3_ were added, followed by incubation for 5 min at room temperature. Then, the solutions were centrifuged at 7,525.71 *g* for 10 min at 4°C. To the supernatant, 120 µL of Griess reagent I (1% sulfanilamide diluted in 3N HCl) was added, and after 5 min, 120 µL of Griess reagent II (0.1% naphthylenediamine dihydrochloride diluted in 3N HCl) was added, with subsequent homogenization and incubation for 10 min in the dark at room temperature. Finally, the supernatant was collected and read at 540 nm. Nitrite concentrations were estimated by interpolating the absorbance of the samples with those determined on a standard curve, which was prepared by diluting NaNO_3_ in distilled water and phosphate buffer, obtaining the final concentrations of 140, 70, 35, 17.5, 8.75, 4.37, 2.18, and 0 μmol/L.

### Statistical analysis

For data whose normal distribution was verified, comparisons between two independent groups were made using Student's *t*-test. For data whose homogeneity of variances was verified, comparisons between more than two groups were made by two-way analysis of variance (ANOVA), followed by Sidak's *post hoc* test. In both cases, the results are reported as means±SE.

When a violation of variance homogeneity was found, the Mann-Whitney test was used for comparisons between two groups, while the Kruskal-Wallis test, followed by Dunn's post-test, was used for comparisons between three or more groups. When comparisons were made by non-parametric tests, data are reported as median and interquartile ranges (25-75%).

Values of P≤0.05 were considered indicative of a statistically significant difference. All analyses were performed by IBM SPSS 21 software, 2012 (IBM Corp., USA).

## Results

### AIA characterization

**Figure 1:**
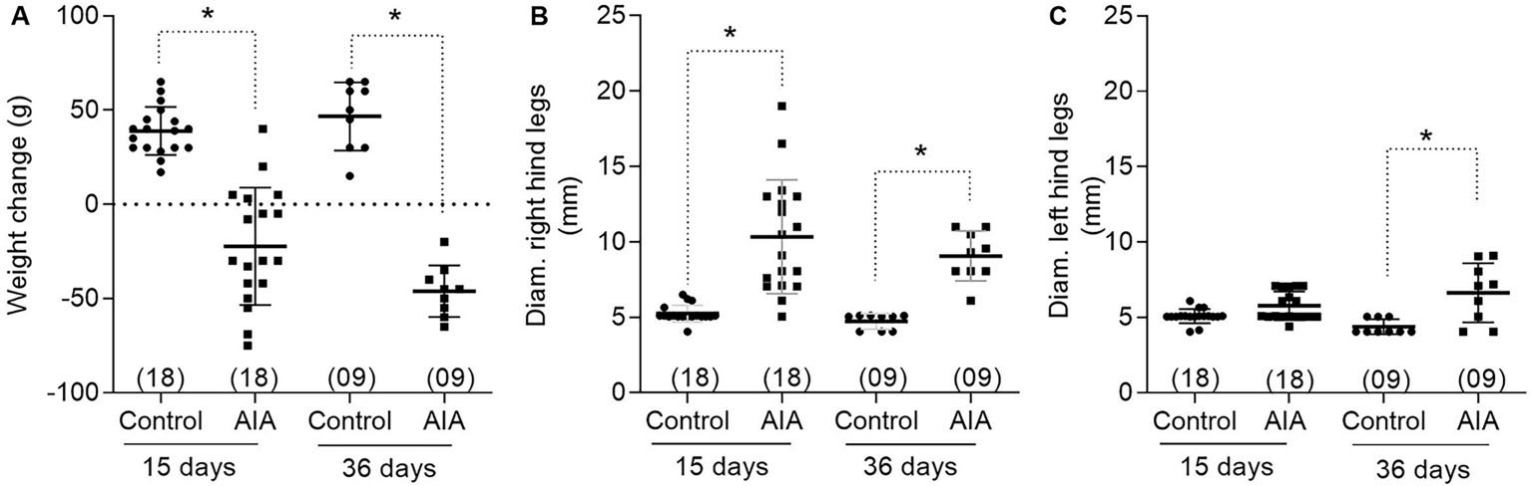
Weight change (**A**), calculated by subtracting the weight on the day of euthanasia from the weight on the day of induction, and the diameter of the right (**B**) and left (**C**) hind legs of control and adjuvant-induced arthritis (AIA) animals, 15 and 36 days after induction/false induction. Horizontal lines represent the median and interquartile range (25-75%). In parentheses is the number of animals in each group. *P<0.05, compared by Kruskal-Wallis test, followed by Dunn's post-test.

AIA animals lost weight during the development of the model. AIA promoted an increase in the diameters of the right and left hind legs. This increase in diameter was quite intense on the right side from day 15 post-inoculation, while in the contralateral side, this increase was observed on day 36 post-induction (Figure 1).

### Morphometric and stereological analysis

**Figure 2:**
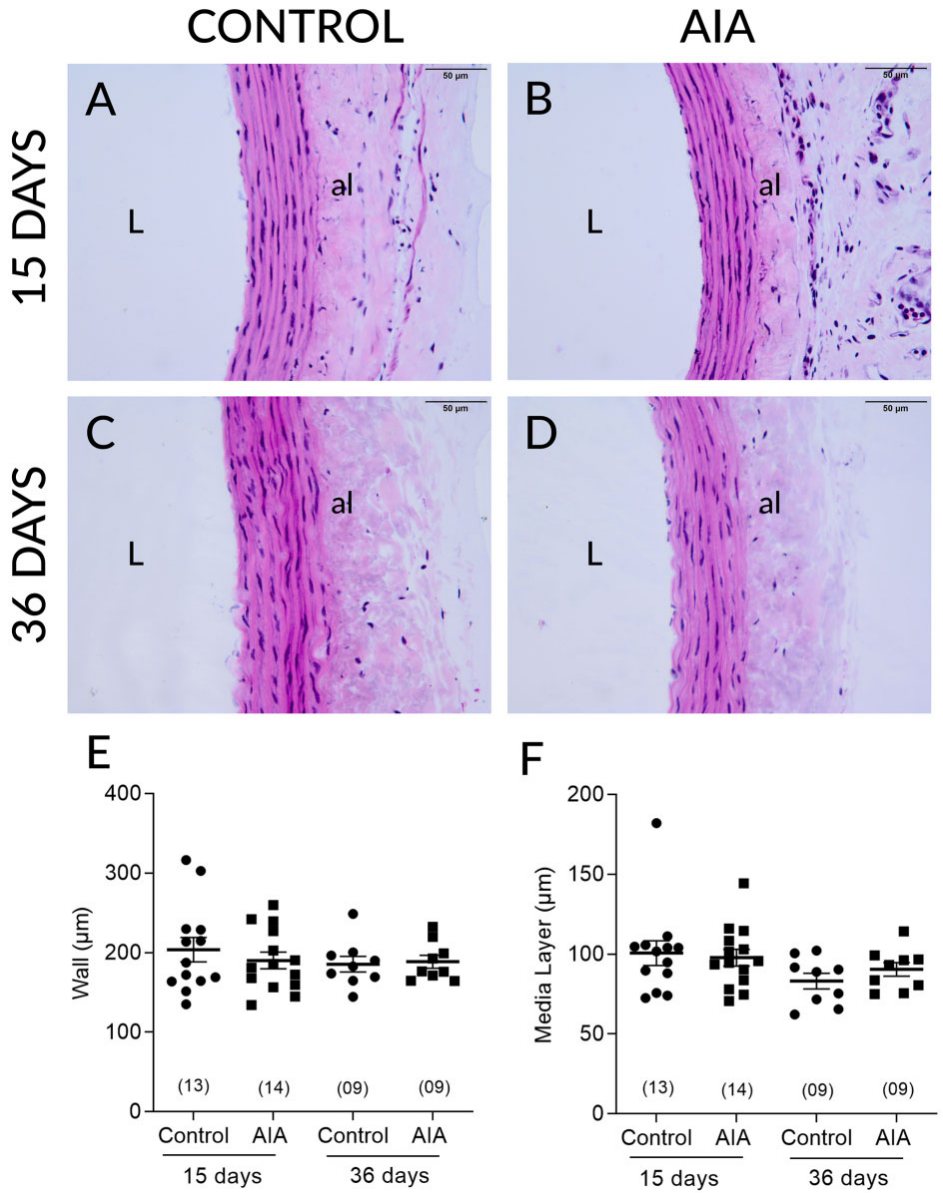
Representative photomicrographs (slides stained with hematoxylin and eosin; scale bar=50 µm) of aortic walls obtained from control and adjuvant-induced arthritis (AIA) animals at 15 (**A** and **B**) and 36 days (**C** and **D**) after induction/false induction and respective thicknesses (µm) of the total wall (**E**) and media layer (**F**). al: adventicia layer; L: lumen. Data are reported as means±SE (two-way ANOVA followed by the Sidak's *post hoc* test). In parentheses is number of animals in each group.

**Figure 3:**
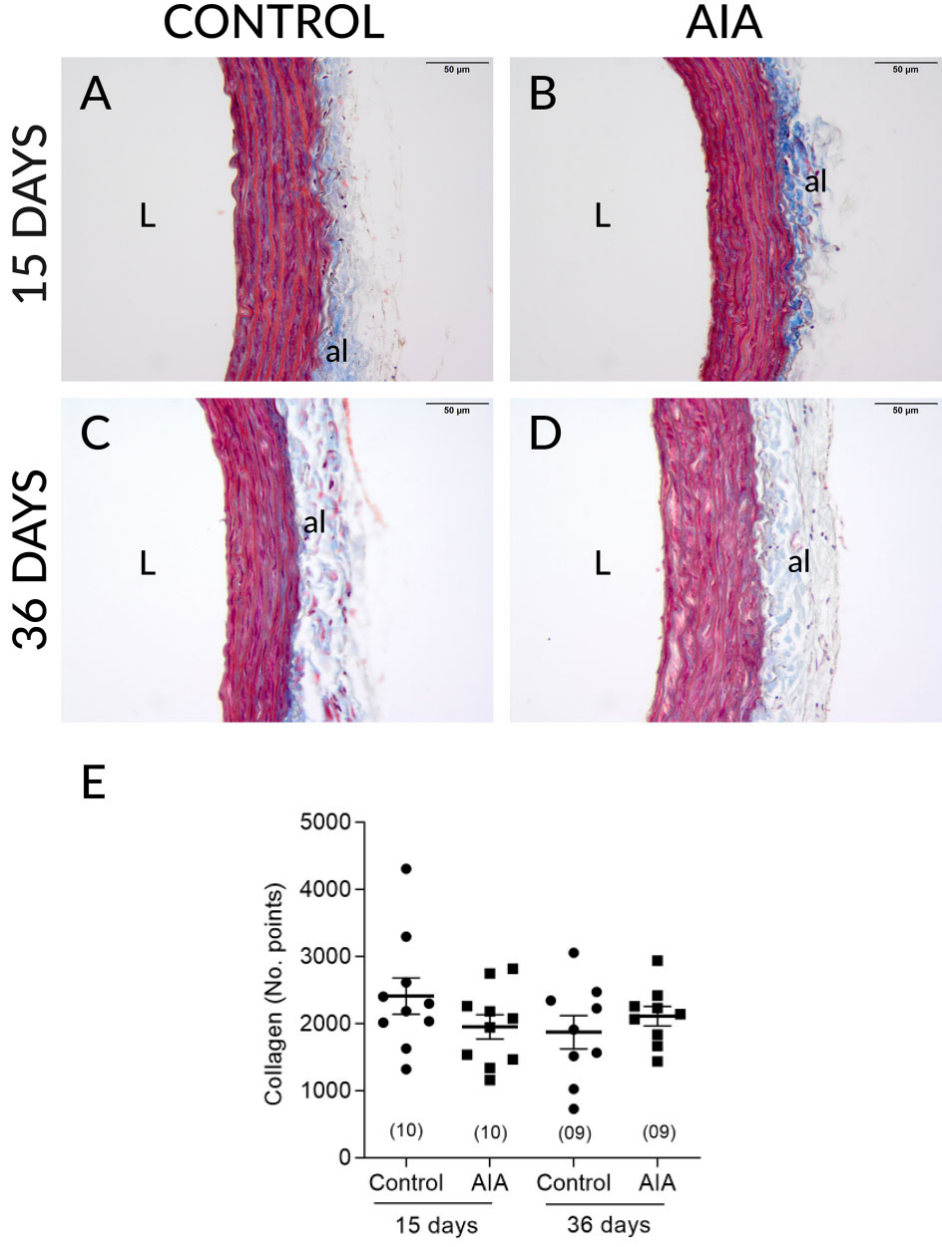
Photomicrographs (Masson trichrome stained slides; total collagen highlighted in blue; scale bar=50 µm) representative of the thoracic aortas of control and adjuvant-induced arthritis (AIA) animals at 15 (**A** and **B**) and 36 days (**C** and **D**) after induction/false induction and respective collagen fiber count values (**E**). al: adventicia layer; L: lumen. Data are reported as means±SE (two-way ANOVA followed by the Sidak's *post hoc* test). In parentheses is number of animals in each group.

AIA did not change the total thickness of the aorta (Figure 2A-E), nor the thickness of the media layer (Figure 2F) at any of the time points analyzed. AIA also did not modify the total number of collagen fibers in the thoracic aorta in either of the two analyzed periods (Figure 3E).

### Study of vascular responsiveness

**Figure 4:**
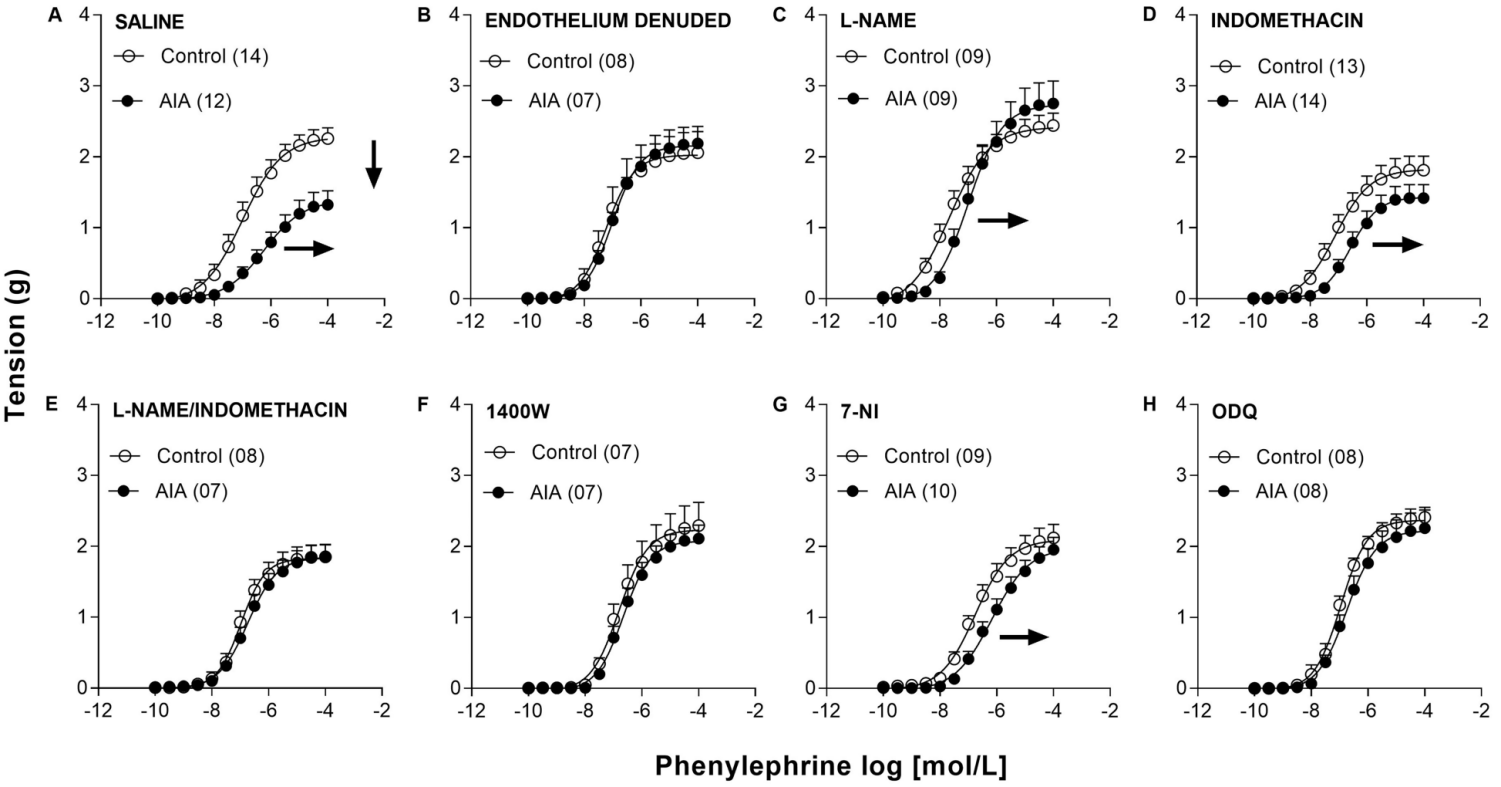
Concentration-response curves for phenylephrine determined in isolated thoracic aorta preparations obtained from animals of the Control and adjuvant-induced arthritis (AIA) groups 15 days after induction/false induction, endothelium-intact (**A**), or denuded (**B**) stimulated by vehicle or endothelium-intact and stimulated by 10^-4^ mol/L L-NAME (**C**), 10^-5^ mol/L indomethacin (**D**), 10^-4^ mol/L L-NAME + 10^-5^ mol/L indomethacin (**E**), 10^-6^ mol/L 1400W (**F**), 10^-4^ mol/L 7-NI (**G**), or 10^-6^ mol/L ODQ (**H**). Data are reported as means±SE. The last point of each concentration-response curve is equivalent to the maximum contractile responses (Emax). In parentheses is number of animals in each group. Vertical arrow indicates AIA-induced reduction in terms of Emax and horizontal arrows indicate AIA-induced reduction in terms of the negative logarithm (pEC50) of the half maximal effective concentration (EC50) (see Table 1).

AIA attenuated the aortic responses to phenylephrine on day 15 after induction, leading to a reduction in Emax and pEC50 values (Figure 4A; Table 1). This hyporesponsiveness to phenylephrine, however, was not observed in endothelium-denuded preparations (Figure 4B; Table 1).

**Table 1:** Maximal response (Emax) and negative logarithm of the half maximal effective concentration (pEC50) values determined in endothelium-intact or denuded thoracic aorta preparations of the control and adjuvant-induced arthritis (AIA) animals, as well as endothelium-intact in the presence of 10^-4^ mol/L L-NAME, 10^-5^ mol/L indomethacin, 10^-4^ mol/L L-NAME + 10^-5^ mol/L indomethacin, 10^-6^ mol/L 1400W, 10^-4^ mol/L 7-NI, and 10^-6^ mol/L ODQ.

Treatment	Emax (g)		pEC50	
	Control	AIA	Control	AIA
Saline	2.26±0.15 (14)	1.33±0.20†* (12)	7.00±0.20‡ (14)	6.22±0.18§* (12)
Endothelium denuded	2.06±0.37 (08)	2.19±0.17 (07)	7.16±0.14 (08)	7.06±0.11 (07)
L-NAME	2.44±0.17 (09)	2.75±0.31 (09)	7.74±0.22 (09)	7.02±0.12* (09)
Indomethacin	1.81±0.20 (13)	1.42±0.19^†^ (14)	7.03±0.18^‡^ (13)	6.44±0.11§* (14)
L-NAME/ Indomethacin	1.85±0.17 (08)	1.86±0.17 (07)	7.03±0.14 (08)	6.75±0.15 (07)
1400W	2.29±0.33 (07)	2.11±0.19 (07)	6.76±0.16^‡^ (07)	6.62±0.10 (07)
7-NI	2.12±0.19 (09)	1.95±0.17 (10)	6.76±0.16^‡^ (09)	6.10±0.14^§^* (10)
ODQ	2.41±0.14 (08)	2.26±0.26 (08)	7.00±0.14 (08)	6.77±0.11 (08)

Data are reported as means±SE. In parentheses is the number of animals in each group. Statistical analyses were parametric. Significant differences are shown between groups (horizontal) and between treatments (vertical) for P≤0.05, compared by two-way ANOVA, followed by Sidak's *post hoc* test. *P<0.05 compared to the control group, within the same treatment; ^†^P<0.05 compared to L-NAME, within the AIA group; ^‡^P<0.05 compared to L-NAME, within the Control group; ^§^P<0.05 compared to both endothelium denuded and L-NAME, within the AIA group.

The presence of L-NAME completely reversed the AIA-induced reduction in phenylephrine Emax. In parallel, L-NAME promoted an increase in pEC50 of the same magnitude in the control and AIA groups. In this manner, in the presence of L-NAME, the difference of Rmax between control and AIA animals was no longer observed, although it persisted in terms of pEC50 (Figure 4C; Table 1). Furthermore, in the presence of indomethacin, the difference in Emax between control and AIA animals disappeared, but the difference in pEC50 persisted (Figure 4D; Table 1). However, this was not due to the reversal of the hyporesponsiveness induced by AIA, but to a slight reduction in the Emax determined in preparations taken from control animals (Figure 4D; Table 1). In addition, no difference in Emax was observed between preparations from control and AIA animals when analyzed in the concomitant presence of L-NAME and indomethacin. This occurred because the treatment attenuated the reduction of this parameter that had been caused by AIA. The concomitant presence of L-NAME and indomethacin also prevented the reduction of pEC50 due to AIA (Figure 4E; Table 1).

AIA also did not promote a hyporesponsiveness to phenylephrine in preparations incubated with 1400W (Figure 4F; Table 1). The presence of 7-NI, in turn, prevented the reduction of Emax to phenylephrine caused by AIA in the aorta of the animals studied, although the reduction in pEC50 persisted (Figure 4G; Table 1). Furthermore, no hyporesponsiveness to phenylephrine caused by AIA was observed in preparations incubated with ODQ (Figure 4H; Table 1).

**Figure 5:**
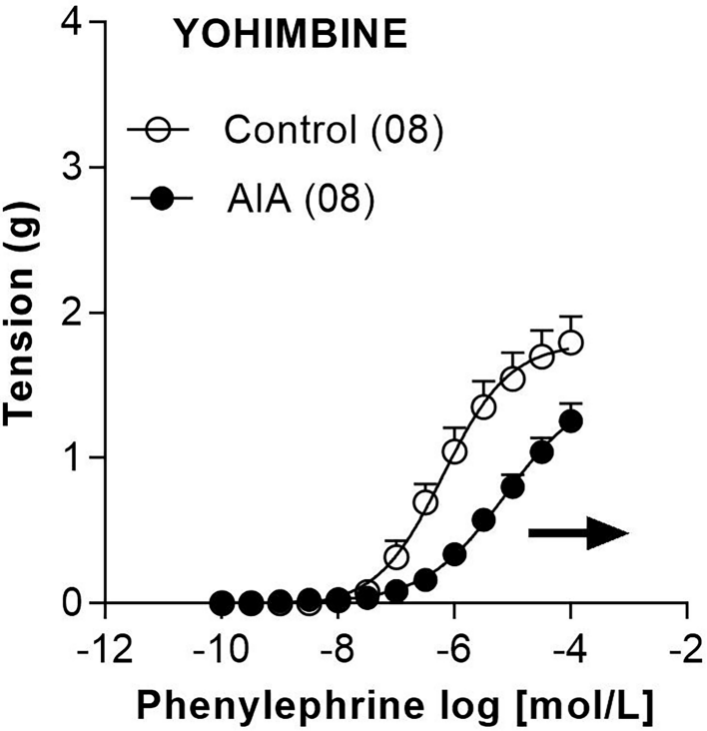
Concentration-response curves for phenylephrine determined in isolated endothelium-intact thoracic aorta preparations of the Control and adjuvant-induced arthritis (AIA) groups in the presence of 10^-6^ mol/L yohimbine 15 days after induction/false induction. Data are reported as means±SE. The last point of each concentration-response curve is equivalent to maximum contractile responses (Emax). In parentheses is number of animals in each group. Horizontal arrow indicates AIA-induced reduction in terms of the negative logarithm (pEC50) of the half maximal effective concentration (EC50).

The presence of yohimbine slightly reduced the responses to phenylephrine in preparations obtained from both control and AIA animals. This reduction of responses occurred in terms of both Emax (from 1.80±0.18 g to 1.26±0.12 g; n=8) and pEC50 (from 6.15±0.14 to 5.09±0.18; n=8). However, despite these slight modifications in response to phenylephrine imposed by treatment with yohimbine, differences in terms of pEC50 between the control and AIA groups persisted (Figure 5).

Finally, AIA also did not alter contractile responses to 0.12 mol/L KCl in either endothelium-intact (Control=1.14±0.13 g; n=8 and AIA=0.90±0.12 g; n=8) or denuded aortas (Control=1.04±0.26 g; n=8 and AIA=1.02±0.23 g; n=8).

### Evaluation of local nitric oxide production

**Figure 6:**
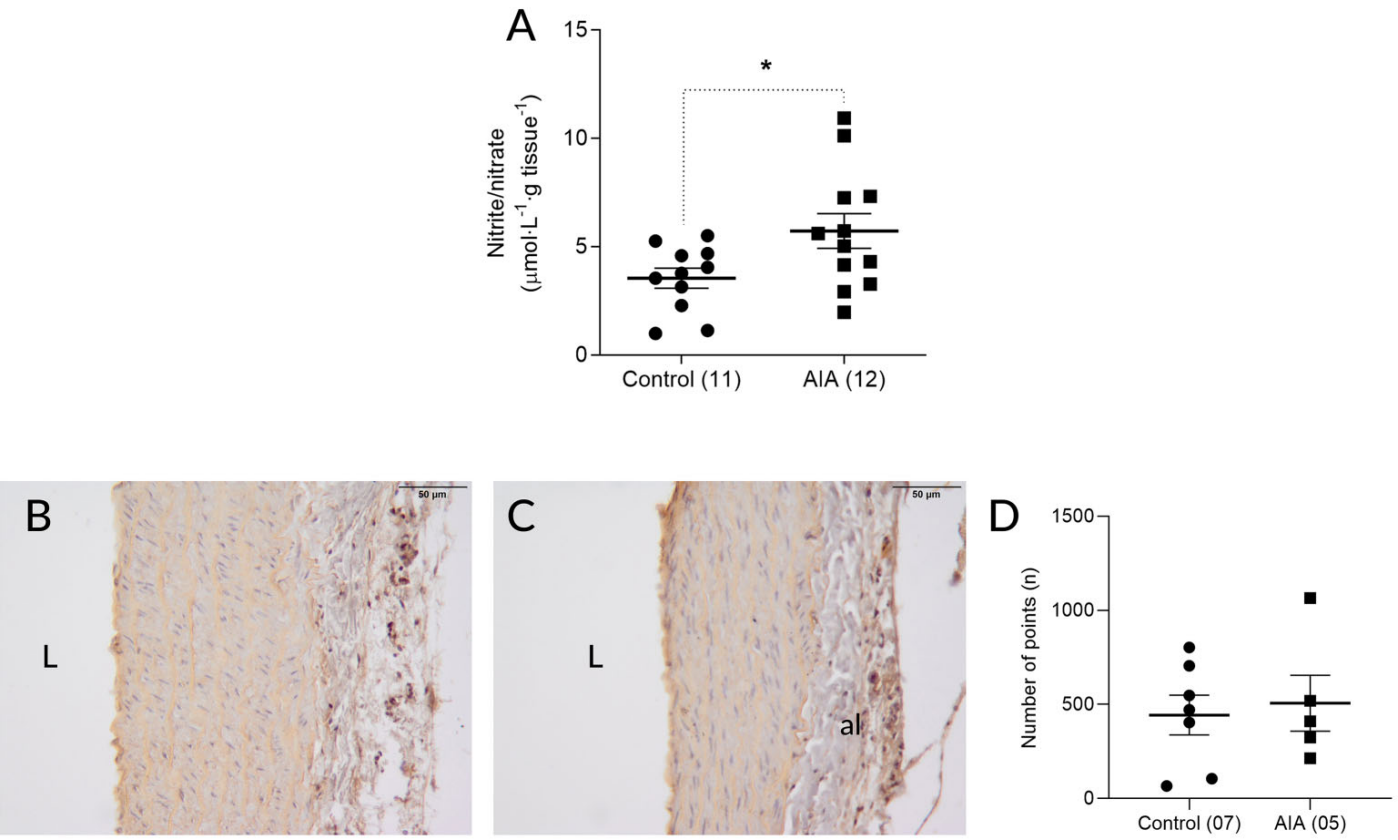
Nitrite/nitrate concentration in thoracic aorta macerates obtained from the control and adjuvant-induced arthritis (AIA) groups 15 days after induction/false induction (**A**). Photomicrographs of histological fields of the thoracic aortic wall of the control (**B**) and AIA (**C**) groups 15 days after induction/false induction with inducible nitric oxide synthase (iNOS) immunolabeled and counterstained with hematoxylin (scale bar=50 µm) and the quantification of iNOS immunolabeling in these fields (**D**), characterized by brownish staining. al: adventicia layer; L: lumen. Data are reported as means±SE. *P<0.05, Student's *t*-test. In parentheses is number of animals in each group.

AIA increased nitrite/nitrate concentration in the thoracic aorta of the animals on day 15 after induction (Figure 6A). On immunohistochemistry, iNOS was detected very sparsely in the intima, media, and adventitia layers of the aortas of the control animals (Figure 6B). In animals submitted to AIA, 15 days after induction, a slight increase in iNOS immunolabeling was observed in the intima and media layers, in the underlying portion of the intima (Figure 6C). The quantification of this immunolabeling, however, showed that this slight increase in iNOS expression was not statistically significant (Figure 6D).

## Discussion

Manifestations of RA in the cardiovascular system can compromise the quality of life and reduce the survival of patients (1,5). Endothelial dysfunction is a well-characterized phenomenon in RA patients and can be reproduced in experimental arthritis models. However, the reduction in response to vasoconstrictor agents that may also arise as a result of arthritis has been much less explored. For this reason, we investigated the mechanisms involved in the reduction of contractile actions induced by phenylephrine in the aorta of rats subjected to AIA. We chose to carry out this study on the 15th day post-induction, as there is evidence of reduction in the contractile responses to α_1_-adrenergic agonists during the initial phase of the AIA model (8,9). Thus, the present study shows for the first time the detailed mechanism by which NO participated in the reduction of the contractile response to α-adrenergic agonists.

The reduction in weight gain of animals subjected to AIA, as well as the increase in the diameter of both the right hind paw, in which the *Mycobacterium tuberculosis* was injected, and the left hind paw confirmed the efficacy of the model (13). This increase in the left paw diameter was most evident 36 days after induction. The modifications chosen here to attest to the effectiveness of the model are part of a larger set of pathophysiological changes caused by AIA (14). In addition, AIA reduced aortic responses to phenylephrine 15 days after induction, which seems to be exclusively due to functional mechanisms, because AIA did not modify the thickness of either the total aortic wall or the media layer. The presence of collagen in the aortic tissues was also not modified by AIA in these animals. Considering that the structural changes do not always occur in parallel with the functional changes, because they can take longer to set in and tend to be perennial, we decided to perform the morphometric and stereological analyses also at 36 days after induction. The data obtained, however, rule out structural changes in these aortas also 36 days after induction.

Since the reduction of the contractile response to phenylephrine was observed on the 15th day after induction, when the inflammatory process starts to have a more systemic feature (characterized by the emergence of the inflammatory process in the contralateral joint), we inferred that mediators coming from the inflamed joints could be triggering this phenomenon. As known, TNF-α, IL-1β, and IL-6, whose circulating levels are elevated in animals subjected to AIA (8,15,16), may stimulate iNOS expression (17,18). In this context, the obtained data showed that L-NAME completely suppressed the reduction of aortic response to phenylephrine caused by AIA, suggesting the involvement of NO. The participation of NO was confirmed by the increased concentration of nitrite/nitrate, which are NO metabolites (19), in the macerate of aortas from AIA animals. These findings corroborated previous studies showing elevated plasma concentrations of NO both in animals undergoing experimental arthritis (20) and in humans affected by RA (21,22). It was also shown that L-NAME treatment can prevent the phenylephrine-induced reduction of contractile responses in blood vessels of the knee joint of rats subjected to AIA (11).

Pro-inflammatory mediators coming from arthritic joints could increase the expression of COX-2 and, consequently, the production of prostanoids (23). Furthermore, it has already been observed that NO can stimulate COX and, consequently, stimulate prostanoid production both in mice macrophages (24) and in the cardiovascular system of rats (25). In the present study, however, the involvement of prostanoids was ruled out since indomethacin was not able to reverse the reduced aortic response to phenylephrine caused by AIA. This observation contrasted with previous studies in which the blockade of prostanoid synthesis did restore rat aortic responses to phenylephrine that had been reduced by arthritis (9) or by IL-6 treatment (26). It is true that the difference in response between control and AIA animals was smaller in the presence of indomethacin. However, this was due to an expected COX-independent reduction in contractile responses to phenylephrine in the aortas obtained of control animals (27), but without a noticeable change in the pattern of these responses in the AIA animals.

Once the participation of NO in the reduction in contractile response of the aorta to phenylephrine was evidenced, we moved on to the identification of the NOS isoform involved in the synthesis of this mediator. Both endothelial NOS (eNOS) and neuronal NOS (nNOS) may be constitutively expressed in the endothelium, logically with regional differences (28-30), while iNOS expression can be induced by the action of inflammatory cytokines (17,18,30). Thus, we initially stimulated some preparations with 1400W. In this condition, we observed that the reduction in response to phenylephrine caused by AIA was completely inhibited. This suggested that the NO production in this vessel was through the action of iNOS, as occurred in other studies in experimental models of arthritis (10) and in humans (31,32).

Although the functional results showed the participation of iNOS in the reduced response to phenylephrine, this was not corroborated by immunohistochemical analysis. Although slightly increased iNOS immunolabeling was observed in the aortas of the AIA animals, this difference was not statistically significant. It is worth noting, however, the absence of statistical significance does not rule out the participation of iNOS in this phenomenon, since the use of immunohistochemistry in the quantification of tissue proteins has limitations.

Preparations were also stimulated with 7-NI. In this condition, we observed a partial reversal of the reduction caused by AIA in aortic responses to phenylephrine. This reversal, although partial, suppressed the difference in Emax, but not in pEC50. Based on these data, we cannot rule out that nNOS also contributed to increase NO production in these aortas. Perhaps the expression of nNOS is stimulated even as a response to the inflammatory process that is established in the endothelium of the animals affected by AIA. This may occur to reduce endothelial dysfunction (33), but it contributes to the reduced response to phenylephrine.

It is also worth noting that AIA did not reduce responses to phenylephrine in deendothelialized aortas, suggesting that NO production occurs in the vascular endothelium. Indeed, the endothelium is the first vascular structure to be influenced by proinflammatory mediators from inflamed joints that arrive via the blood. For this reason, it is plausible to assume that the endothelium is also the site where the concentration of these pro-inflammatory mediators, as well as the induction of iNOS stimulated by them, is higher. Moreover, previous studies show that nNOS, which is possibly also involved in the response alteration studied here, is predominantly found in the vascular endothelium (28,29,33,34).

In the presence of ODQ, the reduction of aortic responses to phenylephrine caused by AIA was suppressed. Indeed, it has already been shown that the vascular relaxation promoted by NO occurs mainly through the stimulation of the enzyme guanylate cyclase, which leads to increased cGMP production (35). In this sense, the accumulation of cGMP may reduce the concentrations of intracellular calcium through different mechanisms, leading to the relaxation of vascular smooth muscle (36).

The phenylephrine-induced release of NO in these preparations could be due to collateral (nonselective) stimulation of α2-adrenergic receptors. It is worth noting that stimulation of α2-adrenergic receptors present in the endothelium can lead to NO release (37,38). The involvement of α2-adrenergic receptors, however, was ruled out because the difference in response between the control and AIA groups, mainly in terms of pEC50, persisted in preparations treated with yohimbine. These results corroborated data obtained in rat knee blood vessels showing that the reduced response to phenylephrine caused by intra-articular administration of Freund's Complete Adjuvant does not involve α2-adrenergic receptors (11). It is worth noting that the responses of the aortas treated with yohimbine were lower compared to untreated aortas, regardless of AIA. This possibly occurred due to antagonism of α2-adrenergic receptors present in vascular smooth muscle, where they can exert vasoconstrictor action in parallel to α1-adrenergic receptors (37).

It has also been reported that cytokine released from arthritis-affected joints, in particular IL-1β, can stimulate the secretion of metalloproteinases (MMPs), especially MMP-2 and MMP-9, in endothelial and vascular smooth muscle cells, which may reduce the action of vasoconstrictor agonists (39,40). However, in the present study, aortic responses to KCl were not modified by AIA. Since no reductions in response to phenylephrine were observed in deendothelialized aortas, it was evident that the contractile capacity of these preparations was not impaired by AIA. Thus, the hypothesis of the involvement of MMPs in the reductions in response to phenylephrine studied here was weakened.

The presented data suggested that the effects of arthritis on blood vessels go beyond the reduction of NO bioavailability caused by oxidative stress. In fact, it was demonstrated here that endothelial dysfunction, at least in the early phase of the inflammatory process related to AIA, was not due to a lack of NO but to an excess of this substance. An in-depth understanding of these nuances of arthritis-related endothelial changes is critical to a broader approach to RA. In contrast, the present study had some limitations since it focused on the earliest phase of AIA development and evaluated only the aorta. Thus, studies at other moments in the evolution of the model, as well as in other vascular beds, will bring important contributions to a better understanding of the vascular manifestations of arthritis.

**Figure 7:**
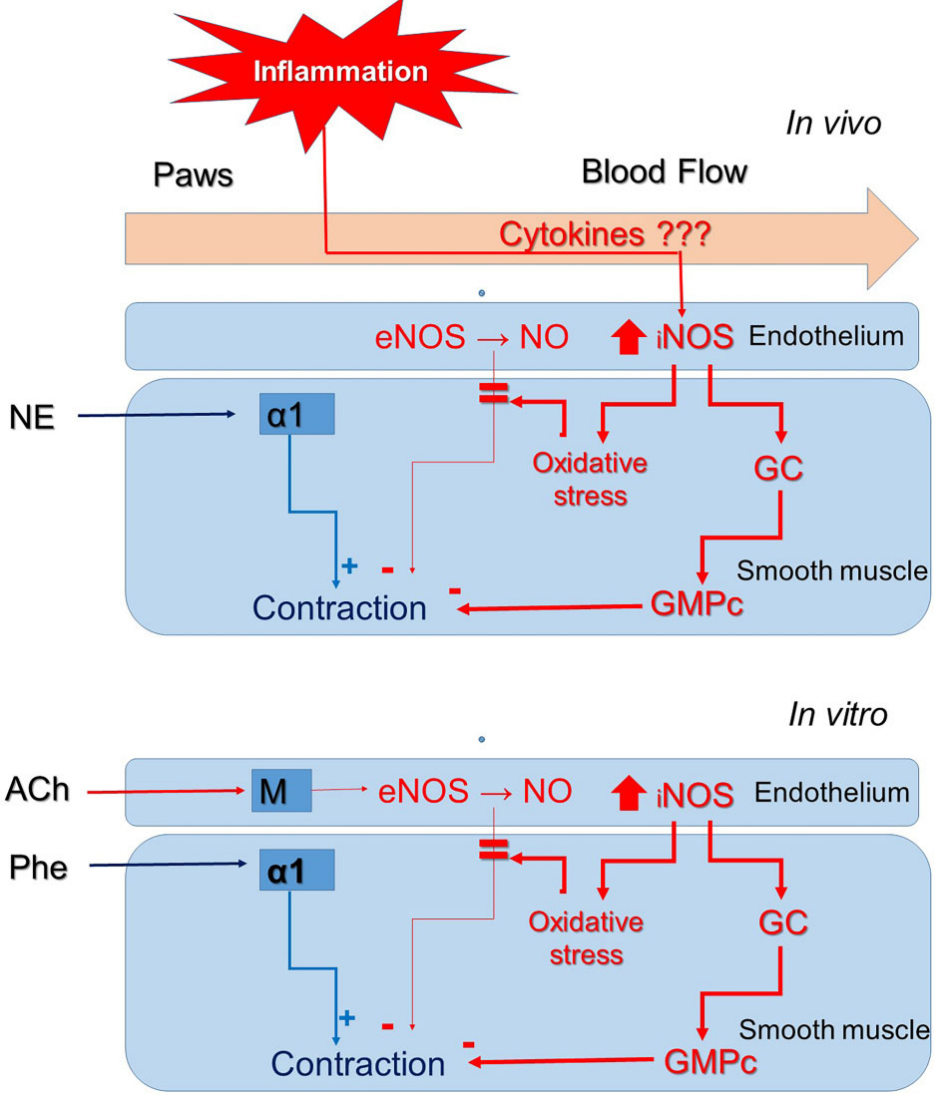
Participation of nitric oxide (NO) in the reduction of aortic responses to phenylephrine (Phe) caused by adjuvant-induced arthritis. *In vivo*, proinflammatory cytokines originating from the joints affected by adjuvant-induced arthritis (AIA) reach the aorta through the circulation where they induce the expression of inducible nitric oxide synthase (iNOS) in endothelial cells and the consequent increase in NO production. The produced NO diffuses to the smooth muscle cells of the medial layer where it activates the enzyme guanylate cyclase (GC), promoting increased cGMP, which in turn attenuates the contraction of the musculature triggered by stimulation of α1-adrenergic receptors by norepinephrine (NE) *in vivo* or by phenylephrine *in vitro* (organ bath). In addition, overexpressed iNOS activity can also lead to a state of oxidative stress that results in reduced bioavailability of NO produced by endothelial NOS (eNOS). It is noteworthy that, *in vitro*, the action of eNOS is stimulated by the action of acetylcholine (ACh) on muscarinic receptors (M).

We concluded that AIA reduced contractile responses of rat aorta to phenylephrine on day 15 post-induction by increasing NO production, possibly by iNOS present in the endothelium of this vessel. The NO produced there, in turn, diffused to the smooth muscle where it stimulated the enzyme guanylate cyclase, thus counteracting the contractile action mediated by phenylephrine (Figure 7).
